# Gut Microbiota Dynamics in Hibernating and Active *Nyctalus noctula*: Hibernation-Associated Loss of Diversity and Anaerobe Enrichment

**DOI:** 10.3390/vetsci12060559

**Published:** 2025-06-06

**Authors:** Ilia V. Popov, Daria A. Peshkova, Ekaterina A. Lukbanova, Inna S. Tsurkova, Sergey A. Emelyantsev, Anastasya A. Krikunova, Aleksey V. Malinovkin, Michael L. Chikindas, Alexey M. Ermakov, Igor V. Popov

**Affiliations:** 1 Faculty “Bioengineering and Veterinary Medicine”, Don State Technical University, 344000 Rostov-on-Don, Russia; 2 Division of Immunobiology and Biomedicine, Center of Genetics and Life Sciences, Sirius University of Science and Technology, 354340 Sirius, Russia; 3 Academy of Biology and Biotechnology, Southern Federal University, 344090 Rostov-on-Don, Russia; 4 Health Promoting Naturals Laboratory, School of Environmental and Biological Sciences, Rutgers State University, New Brunswick, NJ 08901, USA; 5 Department of General Hygiene, I.M. Sechenov First Moscow State Medical University, 119435 Moscow, Russia

**Keywords:** veterinary microbiology, microbial ecology, wildlife, 16S rRNA, bats

## Abstract

Hibernation in mammals entails profound physiological changes that are known to impact host-associated microbial communities, yet its effects on the gut microbiota of synanthropic bats remain underexplored. In this study, we investigated the gut bacterial composition and diversity of *Nyctalus noctula* before and during hibernation using high-throughput 16S rRNA amplicon sequencing. Fecal samples from individually banded bats were collected under controlled conditions at a rehabilitation center and analyzed for alpha and beta diversity, as well as differential taxonomic abundance. Hibernation was associated with a marked reduction in microbial diversity according to the Shannon and Simpson indices and a distinct restructuring of gut communities based on the Bray–Curtis dissimilarity index. Active bats exhibited a diverse microbiota enriched in facultative anaerobes, including *Lactococcus*, *Enterococcus*, and *Escherichia*–*Shigella*, while hibernating individuals were dominated by obligate anaerobes, such as *Romboutsia* and *Paeniclostridium*. These findings suggest a contraction and functional specialization of the gut microbiota during torpor, potentially reflecting adaptations to fasting, hypothermia, and reduced gut motility. Our results demonstrate that the bat’s gut microbiome is highly responsive to physiological status and underscore the importance of microbial ecology for understanding the host’s energy balance and health under seasonal contexts.

## Simple Summary

Bats are important animals in nature and human health, partly because they can carry many different microbes. Some bat species hibernate during winter when their food base is limited. Hibernation involves a lowered body temperature, reduced food intake, and slowed metabolism, all of which may influence gut microbiota. Using high-throughput sequencing techniques, we analyzed fecal samples from individual bats before and during hibernation in rehabilitation center settings. We found that hibernation significantly reduced the overall diversity of gut bacteria. While active bats had a broader range of bacteria, including those associated with digestion and immune function, hibernating bats showed an increased presence of bacteria that thrive without oxygen and specialize in fermenting basic substrates, such as amino acids and simple carbohydrates. These shifts suggest that the bat’s gut microbiota adapts to help the host conserve energy during hibernation. Our findings highlight the dynamic nature of gut microbiota in response to physiological changes and provide insights that may help in understanding microbial roles in animal health, particularly in species that live near humans and may influence the ecosystem and public health.

## 1. Introduction

Bats (Chiroptera) are the second most diverse order of mammals, with more than 1400 species. Moreover, bats represent about 20% of all living mammals worldwide [1,2,3]. These animals play an important role in One Health, mostly as the hosts of various microbes, including emerging ones [4]. These animals are recognized as natural reservoirs for emerging viruses, such as SARS-CoV, MERS-CoV, SARS-CoV-2, Ebola virus, Nipah virus, and Hendra virus [5,6,7]. Some researchers suggest that the emergence of bat-derived pathogens is associated with bats’ unique immune system features, such as a limited inflammatory response to infections, which allows them to co-exist with deadly pathogens for a long time [8]. This feature is accompanied by their ability to fly and their long lifespan compared to mammals of the same size, contributing to the wider spread of microbes in time and space [9,10,11]. Also, urbanization results in the alteration of the bats’ area of habitation, promoting their synanthropic behavior, as their natural sites of roosting are shrinking [12,13,14]. Some studies even show that urban areas are preferable for bats, as the abundance of food, the absence or low prevalence of predators, and the dense arrangement of buildings suitable for roost sites create a beneficial environment for bats [15,16,17]. Thus, all the above emphasize the need to study the microbiome of bats from the One Health perspective, although it should be mentioned that the viruses these animals carry are studied more deeply in comparison to other microbial communities, which is associated with the greater influence of bat-borne viruses on healthcare and domestic animal welfare [9]. Nevertheless, the investigation of bat-associated bacteria and other microorganisms is also important for biomedical science, as bats harbor ancestral lineages of pathogens, spread antimicrobial resistance genes, and carry bacteria, which are of interest for biotechnological applications [18]. Thus, all of the above emphasizes the need for a deeper investigation of bats’ microbiomes.

Some bat species, specifically insectivorous ones, hibernate during the winter season. Hibernation in mammals is generally characterized by food deprivation, decreased metabolic activity, a reduced body temperature, and alterations in the immune system [19,20,21,22]. All the above, in one way or another, contribute to the mammals’ gut microbiota. However, the specific shifts of gut microbiota composition and diversity associated with hibernation in bats are still poorly studied. Since synanthropic bat species hibernate in areas where humans and domestic animals live, the lack of data regarding microbiota in hibernating bats poses a great threat to One Health.

Building on our earlier culture-dependent survey that documented hibernation-related shifts in the cultivable gut microbiota of *Nyctalus noctula*, an insectivorous species abundant in urban environments [23], we now extend the analysis to the culture-independent method of bacterial community analysis. This transition is ecologically informative, as culture-dependent techniques detect only a fraction of microbial diversity, often missing fastidious or unculturable organisms. In contrast, high-throughput sequencing approaches provide a comprehensive, unbiased view of the microbiota, enabling the detection of rare or uncultured taxa and offering deeper ecological insights [24,25]. Here, we apply high-throughput 16S rRNA amplicon sequencing to individually banded *N. noctula* sampled before and during torpor induced within rehabilitation center conditions to quantify hibernation-associated changes in alpha and beta diversity and identify bacterial taxa whose differential abundance may underpin host energy management.

## 2. Materials and Methods

### 2.1. Sampling

Sampling for microbiota analysis was conducted at the Rostov Bat Rehabilitation Center at Don State Technical University, as has been described in our previous studies [23,26]. We collected fecal samples from 19 *N. noctula* in an active (October 2023) and hibernating (March–April 2024) state. Bats were collected by volunteers of the Bat Rehabilitation Center between September and October 2023 in response to requests from residents of Rostov-on-Don, who reported the presence of bats in their households. Following their transfer to the rehabilitation center, all animals underwent veterinary examinations, and only individuals deemed clinically healthy were included in the study. All bats included in this study were males and assessed to be of reproductive age, neither juvenile nor senescent. Age estimation was based on fur coloration, dental conditions, and general physical status, including body weight and wing membrane integrity. The diet of bats in the rehabilitation center consisted of mealworms and superworms. Each bat included in the study was banded, and sampling was conducted from each bat individually. Fecal samples for both active and hibernating states were collected from the same individuals to allow for paired comparisons of microbiota changes within subjects. During the active state, bats were kept under a natural light cycle and ambient humidity conditions ranging from 40% to 60%. Hibernation was induced by placing boxes with bats in a dark environment at a constant temperature of 10 °C with relative humidity maintained around 80%. A minimum of 0.5 g of fecal samples was collected from each bat, placed in sterile 2 mL tubes with 1 mL of IntactRNA (Evrogen, Moscow, Russia), and frozen at −80 °C after overnight incubation at room temperature.

### 2.2. High-Throughput 16S rRNA Sequencing and Bioinformatic Data Processing

DNA from the fecal samples was extracted with the SKYAmp Stool DNA Kit (SkyGen, Moscow, Russia). The concentration of input DNA used for library preparation was at least 20 ng/µL, as measured with a Qubit 3.0 fluorometer using the dsDNA High Sensitivity (HS) Quantitation Kit (Thermo Fisher Scientific, Waltham, MA, USA). V3-V4 16S rRNA library preparation was conducted with the Quick-16S NGS Library Prep Kit (Zymo Research, Irvine, CA, USA) according to the manufacturer’s protocols. The quality and amplification performance of the prepared libraries were assessed via real-time PCR during the targeted sequence amplification and barcode addition steps, as described in the kit protocol. Primers used for the amplification of V3-V4 regions of the 16S rRNA gene were 341F (CCTACGGGDGGCWGCAG, CCTAYGGGGYGCWGCAG) and 806R (GACTACNVGGGTMTCTAATCC). PCR was performed using 20 cycles for the targeted amplification and 5 cycles for the barcode addition, following the manufacturer’s recommendations. The final library was sequenced on Illumina MiSeq with a v3 Reagent Kit (600 cycles) used according to the manufacturer’s instructions (Illumina, San Diego, CA, USA).

The quality of the raw reads in the FASTQ format was assessed with the fastqc (version 0.12.0) software. Reads that passed quality control were analyzed using the QIIME2 (version amplicon-2024.5) software [27]. Denoising was performed with the Deblur pipeline implemented in QIIME2 [28]. Diversity metrics were also calculated using acquired amplicon sequence variant counts based on Shannon [29], Simpson [30], and Pielou [31] indices for alpha diversity and the Bray–Curtis dissimilarity index [32] for beta diversity. The taxonomic classification of amplicon sequence variants was conducted using the 16S rRNA Silva database, limited to V3-V4 regions, as a reference [33,34].

### 2.3. Statistical Analysis

Statistical analysis of microbiota data was conducted using the programming language R v4.4.1 (R Foundation for Statistical Computing, Vienna, Austria). Differences in alpha diversity indexes were determined with the Mann–Whitney test [35]. Beta diversity was compared with the permutational multivariate analysis of variance (PERMANOVA) [36] with the number of permutations set to 999 within the adonis function from the vegan package (version 2.6–6.1) [37]. Differential abundance analysis was performed with the MaAsLin2 package (version 1.7.3) [38]. The results of hypothesis testing were adjusted with the Benjamini–Hochberg false discovery rate. Adjusted *p*-values were considered significant at *p* < 0.05. The statistical analysis results were visualized with the ggplot2 package (version 3.5.1) [39].

## 3. Results

### 3.1. Gut Bacterial Diversity

**Figure 1:**
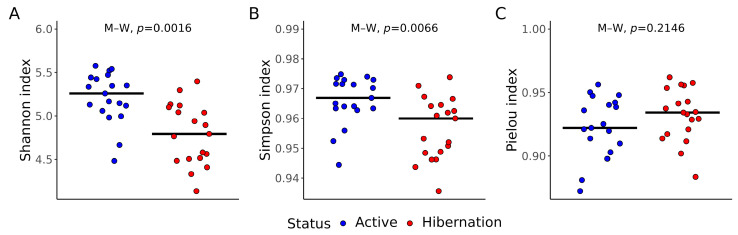
Alpha and beta diversity of gut bacterial communities in active and hibernating *Nyctalus noctula* (*n* = 19). Jitter plots represent differences in alpha diversity based on (**A**) Shannon, (**B**) Simpson, and (**C**) Pielou indexes. M–W = Mann–Whitney test.

Gut bacterial diversity was significantly altered by hibernation in *N. noctula*. There was a significant decrease in Shannon (*p* = 0.0016) and Simpson (*p* = 0.0066) indices in the hibernating bats in comparison to the active animals of the same species (Figure 1A,B). However, according to the Pielou index, no significant differences (*p* = 0.2146) existed in the evenness of the studied gut microbial communities (Figure 1C).

**Figure 2:**
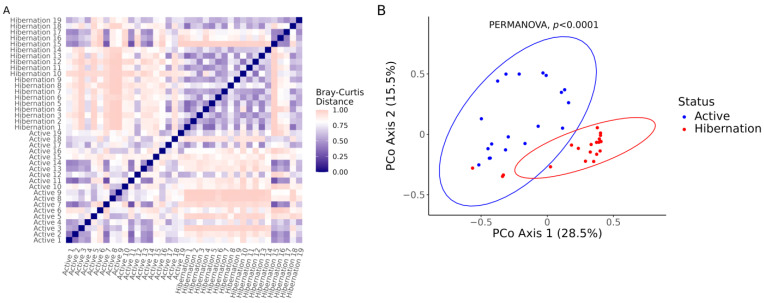
Beta diversity of gut bacterial communities in active and hibernating *Nyctalus noctula* (*n* = 19). (**A**) Heatmap of Bray–Curtis dissimilarity indices between samples, visualized as a symmetric matrix. Samples are ordered by physiological status (Active or Hibernation), with darker colors indicating greater similarity (lower distance). (**B**) Principal Coordinate Analysis (PCoA) plot based on Bray–Curtis distances. Each point represents a sample, colored by status (blue for Active, red for Hibernation). Ellipses represent 95% confidence intervals for each group. PERMANOVA = permutational multivariate analysis of variance.

The distance matrix based on the Bray–Curtis dissimilarity index revealed a clear clustering of microbial communities by physiological status, with lower intra-group dissimilarities and higher inter-group dissimilarities (Figure 2A). Hibernating bats exhibited more tightly clustered communities compared to the more variable communities observed in active bats, suggesting a homogenization of gut microbiota during hibernation. PCoA based on the Bray–Curtis distances supported this observation, showing a distinct separation between active and hibernating groups along the first two principal coordinates (Figure 2B). The first two axes explained 28.5% and 15.5% of the total variance, respectively. This separation was statistically significant as determined by PERMANOVA (*p* < 0.0001).

### 3.2. Gut Bacterial Communities Composition

**Figure 3:**
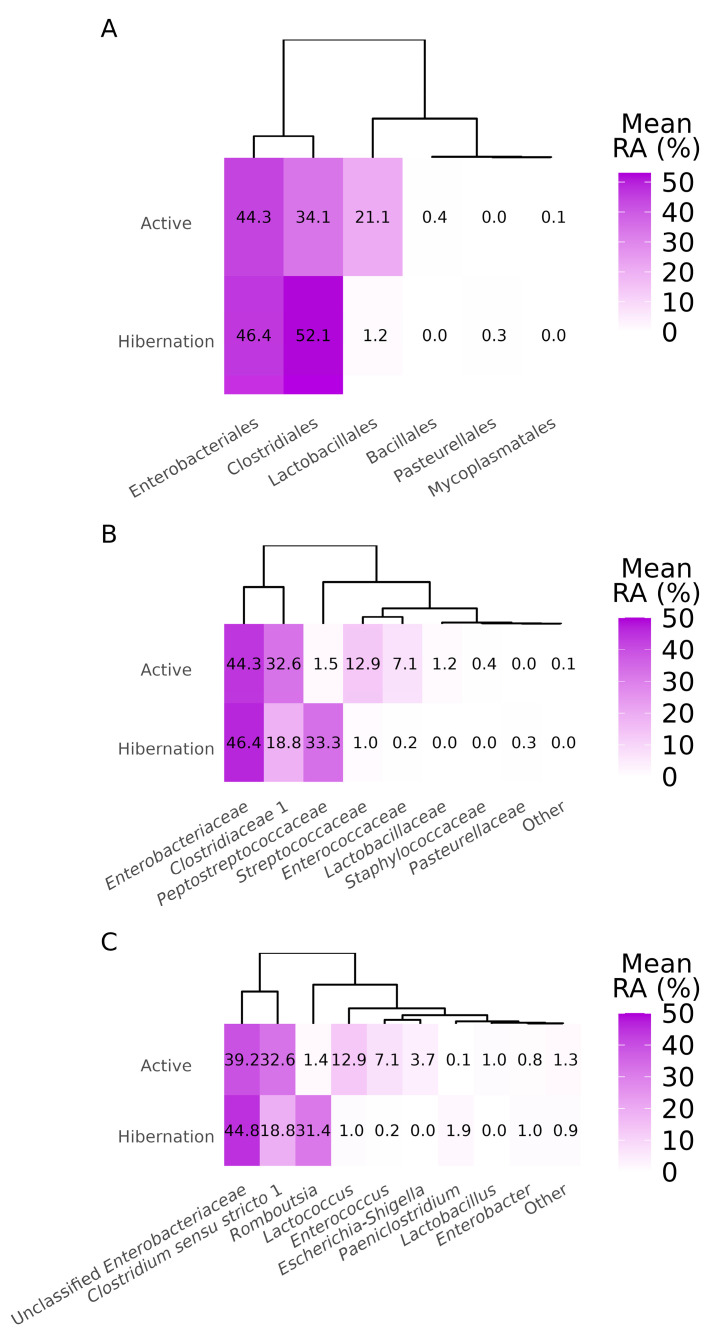
Taxonomic composition of gut bacterial communities in active and hibernating *Nyctalus noctula* (*n* = 19). Heatmaps display the mean relative abundance (RA, %) of the most abundant taxa at three taxonomic levels: (**A**) order, (**B**) family, and (**C**) genus. Dendrograms represent the hierarchical clustering of taxa based on their abundance profiles. Warmer colors indicate higher relative abundances. RA = relative abundance.

Metataxonomic profiling of the gut microbiota in *N. noctula* revealed distinct differences in microbial community composition between active and hibernating states. At the order level, both groups were dominated by Enterobacteriales and Clostridiales. However, hibernating bats exhibited a marked increase in Clostridiales (52.1%) and a concurrent reduction in Lactobacillales (1.2%) compared to active animals (34.1% and 21.1%, respectively) (Figure 3A).

Family-level comparisons further demonstrated a shift in dominant taxa: while *Enterobacteriaceae* remained predominant in both groups, active bats harbored elevated levels of *Streptococcaceae* (12.9%), *Enterococcaceae* (7.1%), and *Lactobacillaceae* (1.2%), which were nearly absent in hibernating individuals (Figure 3B). In contrast, the relative abundance of *Peptostreptococcaceae* markedly increased in hibernating bats (33.3% vs. 1.5% in active animals).

At the genus level, active bats exhibited higher relative abundances of *Lactococcus* (12.9%), *Enterococcus* (7.1%), *Escherichia*–*Shigella* (3.7%), and *Lactobacillus* (1.0%). In contrast, hibernating bats were enriched in *Romboutsia* (31.4%) and *Paeniclostridium* (1.9%) (Figure 3C).

**Figure 4:**
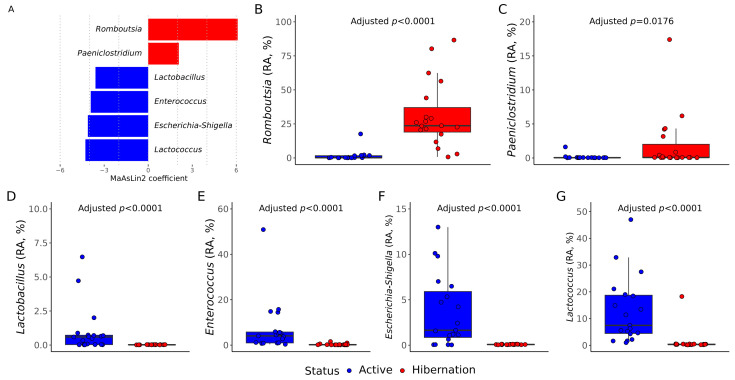
Differentially abundant genera between active and hibernating *Nyctalus noctula* (*n* = 19) were identified using MaAsLin2. (**A**) Barplot of taxa significantly associated with hibernation status based on MaAsLin2 coefficients (*p* < 0.05). Positive values (red) represent enrichment in hibernating bats; negative values (blue) represent enrichment in active bats. (**B**–**G**) Boxplots showing relative abundance (RA, %) distributions of key taxa significantly differing between groups: *Romboutsia*, *Paeniclostridium*, *Lactobacillus*, *Enterococcus*, *Escherichia*–*Shigella*, and *Lactococcus*.

Differential abundance analysis using MaAsLin2 identified several taxa that significantly differed between physiological states (*p* < 0.05). Hibernation was associated with a significant increase in the relative abundance of *Romboutsia* (*p* < 0.0001) and *Paeniclostridium* (*p* = 0.0176), both belonging to obligate anaerobic Firmicutes (Figure 4A–C). In contrast, active bats showed significantly higher relative abundances of *Lactobacillus*, *Enterococcus*, *Escherichia*–*Shigella*, and *Lactococcus* (*p* < 0.0001), reflecting a shift toward facultative anaerobic and aerotolerant bacteria (Figure 4A,D–G).

## 4. Discussion

This study provides a detailed, culture-independent view of how the gut microbiome of the synanthropic bat *Nyctalus noctula* reorganizes between physiologically active and torpor states. By combining diversity metrics, beta diversity ordinations, and differential-abundance modeling, we show that hibernation is accompanied by a contraction of alpha diversity; strong, status-driven community separation; and a taxonomic shift from aerotolerant and facultative-anaerobic taxa to strictly anaerobic, fermentative lineages in gut bacterial communities of the studied bat species. Together, these results extend earlier culture-based observations in *N. noctula* [23] and correspond to the patterns described for ground squirrels [40], bears [41], and several rhinolophid bats [42,43], suggesting that convergent microbial strategies underpin mammalian hibernation [44].

According to the results of the alpha diversity assessment, Shannon and Simpson indices decreased in hibernating animals, whereas Pielou evenness remained unchanged. Reduced gut bacterial richness has also been documented in *Rhinolophus ferrumequinum* and *R. pusillus* during torpor [42,43] and likely reflects a contraction towards a core consortium resilient to low intestinal throughput, prolonged gut retention time, and host fasting. Results from the beta diversity analysis based on the Bray–Curtis dissimilarity index and PCoA confirmed this contraction: intra-hibernation dissimilarities were lower than intra-active ones, with significant differences in these values between bats with different physiological statuses. Similar “microbiome tightening” has been interpreted as ecological filtering, wherein only taxa able to tolerate hypothermia, nutrient scarcity, and periodic immune suppression persist [44].

Differential abundance analysis revealed that *Romboutsia* and *Paeniclostridium*, both strict anaerobes known for fermenting amino acids and simple carbohydrates [45,46,47], were significantly enriched in hibernating bats. In contrast, *Lactobacillus*, *Enterococcus*, *Escherichia*–*Shigella*, and *Lactococcus*, which are all facultative anaerobes [48,49], were more abundant in active animals. *Lactobacillus*, *Enterococcus*, and *Lactococcus* are classical lactic acid bacteria, commonly used as probiotics or fermentation starters in food production [50,51,52]. *Escherichia*–*Shigella*, on the other hand, includes metabolically versatile gut commensals, some of which can act as opportunistic pathogens [53,54].

These compositional shifts likely reflect physiological adaptations to the radically different metabolic landscapes of hibernation and activity. In the hibernating state, enrichment of obligate anaerobes such as *Romboutsia* and *Paeniclostridium* may support the host’s energy conservation by maximizing fermentation of residual substrates under low intestinal motility and anoxia. Conversely, the higher abundance of facultative anaerobes and lactic acid bacteria during active periods suggests a microbiota optimized for a more oxygenated, nutrient-rich gut environment, potentially enhancing nutrient absorption and immune readiness. The prevalence of taxa like *Escherichia*–*Shigella*, with broad metabolic capabilities, further supports the idea of a versatile microbiome tuned to meet the energetic demands of activity. Also, interestingly, some lactic acid bacteria, particularly belonging to the *Lactobacillus* and *Lactococcus* genera, have been shown to possess pro-mutagenic activity in *N. noctula* gut microbiota [55], suggesting that their enrichment may also influence host signaling beyond the metabolism. Taken together, these findings point to a dynamic, functionally responsive gut ecosystem that mirrors host physiology and plays an active role in energy homeostasis and host–microbiota communication.

Despite the novel insights provided by this study, several limitations should be acknowledged. First is the use of 16S rRNA V3-V4 region sequencing, which, while effective for identifying bacterial taxa at the genus level, has limited resolution for taxonomical identification of sequencing data on the species level [56,57]. For example, Commichaux et al. showed that the extension of reference databases for the metataxonomic profiling of amplicon high-throughput sequencing data results in the loss of taxonomic resolution over time [58]. Furthermore, the 16S rRNA sequencing allows for the analysis of only bacterial communities, excluding important microbial groups such as fungi, viruses, and archaea, which may also play crucial roles in gut microbiota dynamics during hibernation. Second, while this study focused on gut microbiota composition, functional analyses of microbial activity were not conducted, leaving open questions about how the observed compositional shifts impact metabolic processes or host health during hibernation. Additionally, the study was conducted on bats housed in a rehabilitation center, where diet and environmental conditions differ from those in the wild, potentially influencing the gut microbiota. The captive diet of studied bats, which consists mainly of mealworms and superworms, does not reflect the diverse insect prey consumed by *Nyctalus noctula* in their natural habitats [59]. This dietary limitation could influence microbiota composition by altering nutrient availability and microbial niches. Also, another important environmental factor is the use of a constant 10 °C temperature during induced hibernation at the bat rehabilitation facility, which does not reflect the natural fluctuations typical of wild conditions. Such variability may influence host physiology and gut microbiota dynamics. The potential impact of captive environments on the gut microbiota is evidenced in some studies comparing bacterial communities of wild and captive bats [60,61]. Although, to our knowledge, no such data are currently available for *Nyctalus noctula*, we recognize that this limitation may affect the generalizability of our findings. Nevertheless, this research provides important baseline data on the taxonomic and ecological structure of bat gut microbiota during hibernation, highlighting conserved microbial responses to torpor and laying the groundwork for future investigations into the functional, immunological, and environmental drivers of these microbial dynamics. Future research would benefit from longitudinal studies, including samples from free-ranging bats, along with multi-omics and in vitro analyses to provide deeper insights into the functional aspects of microbiome changes during hibernation.

## 5. Conclusions

This study provides valuable insights into the gut microbiota composition and diversity of *Nyctalus noctula* during active and hibernating states. Our findings reveal significant shifts in microbial communities between these physiological states, with active bats showing higher alpha diversity and the presence of taxa associated with increased metabolic activity, such as *Enterococcus* and *Lactococcus*. In contrast, hibernating bats exhibited a dominance of *Romboutsia* and *Paeniclostridium*, reflecting microbial adaptations to a low-energy state. These results underscore the dynamic nature of gut microbiota in response to metabolic and environmental changes, and highlight the importance of further investigating microbiome–host interactions in bats, particularly in the context of One Health. Future research should aim to explore the functional implications of these microbial shifts and their potential role in host health and disease transmission during hibernation.

## Data Availability

The raw sequencing data used for this study are available in the National Center for Biotechnology Information’s Short Read Archive under BioProject ID PRJNA1173750.

## Abbreviations

The following abbreviations are used in this manuscript:

16S rRNA	16S Ribosomal Ribonucleic Acid
DNA	Deoxyribonucleic Acid
RNA	Ribonucleic Acid
NGS	Next-Generation Sequencing
PCR	Polymerase Chain Reaction
V3-V4	Variable Regions 3 and 4 (of the 16S rRNA gene)
M–W	Mann–Whitney Test
PERMANOVA	Permutational Multivariate Analysis of Variance
PCoA	Principal Coordinates Analysis
QIIME2	Quantitative Insights into Microbial Ecology 2
R	R Programming Language
